# Dissociable Components of Information Encoding in Human Perception

**DOI:** 10.1093/cercor/bhab189

**Published:** 2021-07-22

**Authors:** Diego Vidaurre, Radoslaw M Cichy, Mark W Woolrich

**Affiliations:** Department of Clinical Medicine, Center for Functionally Integrative Neuroscience, Aarhus University, Aarhus 8000, Denmark; Department of Psychiatry, University of Oxford, Oxford OX37JX, UK; Wellcome Trust Center for Integrative Neuroimaging, University of Oxford, Oxford OX37JX, UK; Department of Education and Psychology, Freie Universität Berlin, Berlin 14195, Germany; Department of Psychiatry, University of Oxford, Oxford OX37JX, UK; Wellcome Trust Center for Integrative Neuroimaging, University of Oxford, Oxford OX37JX, UK

**Keywords:** MVPA, oscillations, stimulus decoding, temporally unconstrained decoding analysis, visual perception

## Abstract

Brain decoding can predict visual perception from non-invasive electrophysiological data by combining information across multiple channels. However, decoding methods typically conflate the composite and distributed neural processes underlying perception that are together present in the signal, making it unclear what specific aspects of the neural computations involved in perception are reflected in this type of macroscale data. Using MEG data recorded while participants viewed a large number of naturalistic images, we analytically decomposed the brain signal into its oscillatory and non-oscillatory components, and used this decomposition to show that there are at least three dissociable stimulus-specific aspects to the brain data: a slow, non-oscillatory component, reflecting the temporally stable aspect of the stimulus representation; a global phase shift of the oscillation, reflecting the overall speed of processing of specific stimuli; and differential patterns of phase across channels, likely reflecting stimulus-specific computations. Further, we show that common cognitive interpretations of decoding analysis, in particular about how representations generalize across time, can benefit from acknowledging the multicomponent nature of the signal in the study of perception.

## Introduction

In recent years, multivariate pattern analysis (MVPA) or decoding analysis on electrophysiological data has emerged as a widely used technique to interrogate when and where in the brain information is processed (Haynes and Rees 2006; Tong and Pratte 2012; Cichy et al. 2014; Grootswagers et al. 2017; Kragel et al. 2018; Vidaurre, Woolrich, et al. 2019; Carlson et al. 2019; Takacs et al. 2020). MVPA is typically used with the objective of maximizing the prediction of information, so that it is not always transparent what aspects of the data are responsible for the significant predictions. However, electrophysiological data have diverse components, such as ongoing oscillations with amplitude and phase modulations in different frequencies (Steriade 2001; Buzsáki and Draguhn 2004; Buzsáki et al. 2013), ultra-slow 1/f drifts of activity and slow cortical potentials (Linkenkaer-Hansen et al. 2001; He 2014), non-oscillatory changes in signal amplitude (Jones 2016; van Ede et al. 2018; Quinn et al. 2021), cross-frequency coupling (Jensen and Colgin 2007; Canolty and Knight 2010), and high-frequency burst events (Buzsáki and Lopez da Silva 2012; Lundqvist et al. 2016). Data-driven decoding methods can incorporate all or some of these elements, but it is unclear which ones are relevant and how. Because these elements are thought to underpin distinct brain mechanisms, knowing which of them are important for prediction would provide important theoretical information about human brain function.

Although we aim at addressing general principles, we here focused on visual processing, whose various levels of processing (e.g., from low-level features to the recognition of semantic content) are known to involve frequency-specific oscillations (Melloni et al. 2007; Kayser et al. 2012; Clarke et al. 2013, 2018; Jensen et al. 2014). Using MEG data recorded while participants viewed object images, and focusing on slow frequencies, we separated task evoked responses into just two components: a non-oscillatory (ultra-slow) and an oscillatory component. We demonstrated the distinct contribution of these components to the results of MVPA, that is, the decoding accuracy for prediction of which image was presented to the participant. In addition, isolating the oscillatory component allowed us to extract stimulus-locked phase information, confirming that the phase of the oscillatory component contains critical information about which image category is being processed by the brain (Rodriguez et al. 1999; Sauseng and Klimesch 2008). Crucially, we showed that stimulus-locked phase information can be predictive of the image category in two different ways: through differences in the latency of the response that are common across channels; and through relative phase differences between channels, to which single-channel analyses like event-related potentials/fields (ERP/F; Pfurtscheller and Lopes da Silva 1999) are blind. We argue that these two (coexisting) phase-related features potentially speak to different physiological mechanisms, and that the dissociation of these components provides a more accurate interpretation of commonly observed phenomena in the analysis of perception—in particular with regards to how the stimulus representations generalize or change across time (as described, for example, using the temporal generalization matrix approach; King and Dehaene 2014).

In summary, we show that the dissociation of these different elements of the data provides a more accurate interpretation of commonly observed phenomena in the analysis of perception, providing a better understanding on how multivariate phase information can encode different visual contents in electrophysiological data.

## Materials and Methods

### Basic Theoretical Background

Conventional MVPA and event-related potentials/fields (ERPs/ERFs), which represent the average pattern of the signal locked to the presentation of the stimulus (Pfurtscheller and Lopes da Silva 1999), are both based on assuming consistent timing (or phase) over trials. This is because conventional MVPA uses the same decoder over all trials at the same time-point within each trial, and ERP/ERF approaches average over trials at the same time-point within each trial. The most fundamental differences between these two approaches relies on MVPA being prediction-based (i.e., decoding-based) and, critically, multivariate over channels—while ERP/ERF analysis is univariate over channels and encoding-based (Weichwald et al. 2015).

We examined MVPA in the context of the temporal generalization matrix (TGM) approach (King and Dehaene 2014), which extends conventional time-resolved decoding analysis. The TGM, as estimated from magnetoencephalography (MEG) in MVPA approaches, is widely used to assess the dynamics of neural representations. It is a T × T matrix of decoding accuracies (where T is the number of time points in the trial), such that one decoding model is trained per time point and tested on each one of the time points of the trial in a cross-validation fashion. The diagonal of the TGM reflects how well can we can decode information time point by time point, indicating the waxing and waning of the different stages of stimulus processing. The off-diagonal of the TGM shows how well decoding models generalize to time points different to those where they were trained; and, therefore, is argued to reflect the stability of the neural code for the neural representation under study. Thus, the off-diagonal elements of the TGM are often interpreted as being related to memory in the most basic definition of the term; that is, the persistency of information and meaning in the brain for longer than an instant. Being data-driven and designed to maximize decoding accuracy, however, the construction of the TGM is not explicitly concerned by the idiosyncrasies of electrophysiological data, for example by how exactly oscillations relate to the prediction. This is in contrast with the extensive body of research on the specific role of oscillations in cognition and information representation (Buzsáki and Draguhn 2004).

### Signal Processing

Data were downsampled to 250 Hz, and low-pass filtered under 10 Hz in order to narrow down the analysis on the lower frequencies. We discarded higher frequencies in order to focus on one single oscillation. Note that focusing on these aspects of the data does not at all imply that the left-out features (e.g., higher frequency oscillations, gamma bursts, cross-frequency interactions, etc.) are not relevant for stimulus processing. Importantly, our conclusions do not lose generality if these left-out aspects also produce significant decoding accuracies.

For each trial and channel, we analytically separated the signal into two uncorrelated components. The first component was computed by applying local regression using weighted linear least squares and a first-degree polynomial model (parametrized to use 100 points, that is, 10% of the trial). This yielded a slow component or trend, which we referred to as non-oscillatory. Note that this component is not oscillatory when considered in 1-s trials, but it could contain traces of ultraslow oscillations in the context of the entire recording. We then regressed out this component onto the original signal. The residual is a detrended oscillation, which we referred to as the oscillatory component. We used this approach to avoid the sinusoidal assumption of Fourier analysis (Huang et al. 2009), which might be less appropriate for the frequencies considered here and the length of the trials (1 s). This not however a critical step in the pipeline, and similar results were obtained with standard Fourier-based filters (now shown).

**Figure 1:**
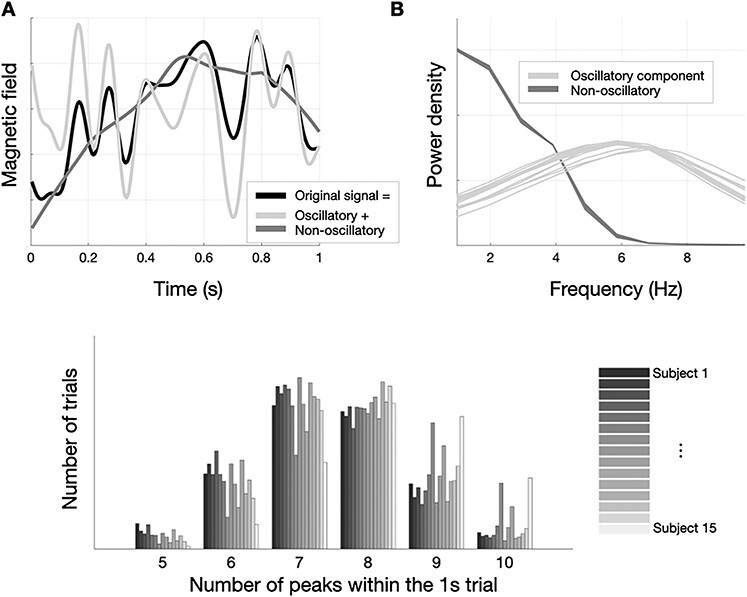
Analytical separation of the signal into oscillatory and a non-oscillatory component. (*A*) Example of the components found in one trial. (*B*) Power of the two components. (*C*) Distribution across subjects of the number of peaks per trial in the oscillatory component (i.e., number of maxima in the signal).

For illustration, Figure 1*A* shows the original signal (below 10 Hz) and the two components for a single trial and channel, from one subject’s MEG data. Figure 1*B* shows the spectral profile of these two components averaged across channels and participants, with the oscillatory component having a dominant 6-8 Hz frequency and the non-oscillatory component not having any frequency peak. In order to further characterize the oscillatory component, Figure 1*C* depicts the distribution of the number of peaks (in the temporal domain) per 1-s-trial in the oscillatory component, reflecting certain homogeneity across subjects. Note that we refer to oscillatory just as the presence of peaks and troughs, without further requirements, and with no explicit consideration of a specific frequency (see Bullock et al. 2003; Donoghue et al. 2020, for more strict criteria and considerations about the oscillatory behavior of brain signals).

### Methods for Standard Decoding Analysis

We used cross-validation to assess the accuracy and cross-generalization accuracy (King and Dehaene 2014) of the decoding for each pair of stimuli. Given a set of trials or repetitions of the experiment, a decoder (regressor or classifier) contains information about how the stimulus is represented in the brain at the time that the decoder was estimated. Mathematically it can be represented as a function $f({x}_t,{y}_t)$, which predicts the stimulus ${y}_t$ from the data ${x}_t$ at time point *t*. Having standardized the data separately for each trial, we used L_2_-penalized linear regression (usually referred to as “ridge regression”; Friedman et al. 2001) as a base decoder, where the predicted variable was encoded as −1 or +1, and we selected the regularization parameter using a nested cross-validation loop. Although logistic regression is more adequate to deal with binary classification, we opted for L_2_-penalized regression because of its lower computational cost, given the high number of stimuli pairs. Note also that standard linear regression, which is intimately related to L_2_-penalized linear regression (since they both minimize the sum-of-squared errors), is equivalent to linear discriminant analysis insofar as the proportions of the classes are equal, as is our case (Friedman et al. 2001).

### Temporally-Unconstrained Decoding Analysis

The principles underlying Temporal Unconstrained Decoding Analysis (TUDA) and standard decoding are similar, except that TUDA uses fewer decoding models (fewer regression parameters) and, in exchange, has a new set of parameters that capture when the decoders activate over time on a trial-by-trial basis. Therefore, the main conceptual difference is that TUDA accounts for between-trial variability in stimulus processing, which standard decoding assumes fixed.

Let us assume that we can represent how the brain encodes information using a set of *K* decoders, that is, functions ${f}_k$that can predict with certain accuracy the value of the perceived stimuli given the brain data. We will also assume that at any given time point of the experiment, there is only one “active” decoder; or, in other words, that every time point was used to train one and only one decoder ${f}_k$. For convenience, let us define an indicator variable ${\gamma}_{tnk}$, such that ${\gamma}_{tnk}=1$ if the *k*-th decoder is active at time point *t* and trial *n*, and ${\gamma}_{tnk}=0$ otherwise.

For standard decoding, the basic premise is that ${\gamma}_{tnk}$ has the same value (either 0 or 1) for all trials $n=1\dots N$. Also, typically, there is a different decoder for each time point $t=1\dots T$, where $T$ is number of time points in each trial. Hence, there are $K=T$ decoders for standard decoding in total.

In the TUDA approach, the key idea is to relax the assumption that ${\gamma}_{tnk}$ has the same value for all trials. In exchange, the number of decoders is considerably lower than with standard decoding (i.e., $K$ is much lower than $T$), so that we are trading temporal flexibility for spatial parameters. While the estimations of the decoders in standard decoding are decoupled from each other, TUDA is a unitary Bayesian (generative) model that includes the regression parameters of the $K$ decoders, the parameters ${\gamma}_{tnk}$, and a transition probability matrix $M$ containing the probabilities of transitioning from one decoder to another within the trials. Similarly to the standard decoding approach described above, the base decoder used for TUDA is a regularized regression model, which makes the results comparable between the two approaches. All the elements of the TUDA model are estimated using variational Bayesian inference (Vidaurre et al. 2016; Vidaurre, Abeysuriya, et al. 2018b).

Although this model was already presented previously (Vidaurre, Myers, et al. 2019), here we applied three modification to improve performance and avoid overfitting. Indeed, overfitting can be a problem of this model if some decoders specialize too much in certain stimulus values. For example, in the hypothetical case that there is an overall shift in the signal when face stimuli are presented as opposed to inanimate objects, there would be the risk that a subset of the decoders takes all time points for “face” trials (by having overall higher regressor parameters), while a separate subset of the decoders takes the other trials (by having overall lower regressor parameters). This degenerate solution is of course not useful to interrogate the cascade of stimulus processing. The improvements we have introduced in this paper are:

1. The decoders are forced to activate in sequence, so that every trial starts with the first decoder ($k=1$) and finishes with the last decoder ($k=K$).
2. The decoders are forbidden to reach out too far in the trial from their mean activation point ($k/T$). In particular, when the decoders are allowed to be active is given by the matrix shown in Supplementary Figure 7.
3. To avoid the aforementioned effect, we constrained the estimation of the decoder parameters so that for all decoders the regression parameters sum up to the same value.

Because the inference of this model can take a bit longer than a standard decoder, we applied TUDA on image “supra”-categories: to whether the image is an animate being, and the size of the represented object. This way, we could also use all trials in the estimation. Also, for computational efficiency, we conducted PCA on the data so that the model was trained on the first 116 principal components (explaining at least 98% of the variance per subject).

### Quantifying Phase-Locking

Given that the distribution of phase across trials in the absence of stimuli is random, we can describe phase-resetting, for each time point after stimulus presentation, as how concentrated is the across-trials distribution of phases at a specific angle. This is commonly measured in practice using the Phase Locking Factor (PLF):${\mathrm{PLF}}_{tj}=\frac{1}{N}\Big|\sum_{n=1}^N{e}^{i\ {\varphi}_{tnj}}\Big|$,where ${\varphi}_{tnj}$ is the instantaneous phase of trial *n* at time point *t* for channel *j* (Tallon-Baudry et al. 1997).

### Permutation Testing

We used permutation testing for both testing the latency of processing and the relative phase between image categories. To test the latency of processing, we took the decoding time courses $\gamma$, which is a 3-D array of dimension $T$ × $N\times K$, where $N$ is the number of trials. For each state (from the second to the last; note that states activate always in a sequence), we collected the exact time at which that state became active. Note that this is not possible for standard decoding, where timings are assumed equal for all trials. We then used permutation testing on the difference in timing between the two types of stimuli (e.g., animate vs. inanimate, or big vs. small). Specifically, we generated surrogated data by permuting the stimulus labels, such that the *P*-value corresponds to the proportion of permuted instances of the data where the difference in timing between the two types of stimuli was higher than in the unpermuted data. If this test turns out to be significant, that would mean that there are different speeds of processing for each image category. This was done for each of the subjects and each of the (K-1) states separately (excluding the first state, which always starts at the first time point of the trial). Tests were combined into a group-level *P*-value using the non-parametric combination algorithm (NPC—Pesarin and Salmaso 2010; Vidaurre, Woolrich, et al. 2019b).

Now, to test if there are cross-channel differences in the relative phase between image categories, we need to factor out the differences in global latency (which were assessed in the previous test). Only then, we will be able to test if the nature of the phase trajectories (i.e., the relative phase between channels at each time point) is distinct across image categories above and beyond how fast these trajectories traverse. We do this by using the temporal realignment provided by the activation times of the decoders, defined by ${\gamma}_{tnk}$; these were used as data-driven time windows containing quasi-stationary stages of processing, that is, with a quasi-stable differential phase pattern. Now, for each of the decoders (i.e., within the decoder-specific time windows), we tested whether the within-class PLF was stronger than the between-classes PLF. We tested this using permutation testing, using, as a base statistic, the PLF across trials for one class plus the PLF across trials for the other class (This sum will be maximized when within-class phase locking dominates between-class phase locking.) By summing this value across channels, we obtained subject-level base statistics; by summing across subjects and channels, we obtained a group-level base statistic. As before, we generated surrogated instances of the data by permuting the stimulus labels, computing *P*-values as the proportion of permutations where the described base statistic was higher than in the unpermuted data. In summary, if phase locking was found to be significantly more consistent within image categories than in general, that would suggest distinct relative phasic patterns across channels for each stimulus condition.

## Code Availability

The custom code to reproduce the analysis can be found in the first authors Github personal site. The code for running TUDA can be found in https://github.com/OHBA-analysis/HMM-MAR.

## Results

We used one of the magnetoencephalography (MEG) data sets presented in Cichy et al. (2016), where 118 different image categories were presented 30 times each to 15 subjects. Presentation lasted 500 ms, and trials were ~1-s long. The multi-channel sensor-space data, epoched around the presentation of each visual stimulus, can be used to train a decoder to predict which visual image is being presented (Cichy et al. 2016).

We reasoned that different aspects of decoding accuracy may separately emerge from different components of the evoked responses. Therefore, we analytically extracted two uncorrelated components from the data: an oscillatory component (with a dominant theta frequency at 6–8 Hz), and a slower, non-oscillatory component (which could be related to very slow oscillations in the context of the entire recording, but not at the single-trial level); see section Materials and Methods for details on the preprocessing and organization of the data, as well as on the decoding methods. See also Figure 1 for an illustration of the separated components. Importantly, even though we focused on a single oscillatory pattern at <10 Hz (predominantly in the theta band; Kayser et al. 2012) in order to convey the messages of the paper more clearly, this does not mean that higher frequencies are not relevant for visual processing; see Supplementary Results for additional analyses on >10-Hz frequencies.

**Figure 2:**
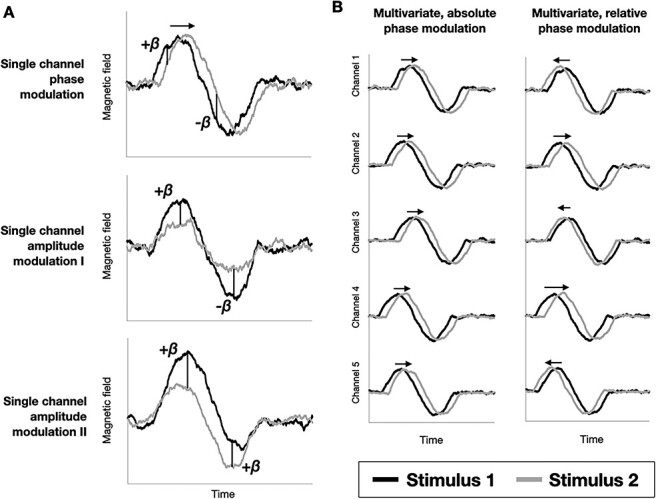
Different types of evoked response differences that can be used by decoding. (*A*) In the univariate setting, evoked response differences could occur either in phase or in amplitude. (*B*) In the multivariate setting, differences in phase could be either global (the phase shift is the same across all channels), or relative (i.e., different, between channels). Arrows represent phase differences in phase-specific panels.

### Oscillations Can Theoretically Contain Different Types of Stimulus-Specific Information

When looking at the oscillatory component, both phase and amplitude modulations can cause differences that can in principle be used by MVPA, when applied time point by time point and across trials on the brain signals. We demonstrate schematically different ways by which an oscillatory component might contain stimulus-specific information, either using single MEG channels at a time, or using multiple channels (i.e., multivariate decoding). This forms the theoretical basis for our subsequent analyses using MVPA on phase in empirical data.

Figure 2*A* illustrates three ways in which differences in evoked responses could cause changes in decoding accuracy between experimental conditions (i.e., here object images) at the single-channel level. The evoked responses (i.e., the ERP/F) for a single channel are depicted for two different stimuli in black and grey. These differences, examined at two time points marked as vertical lines (separated according to the period of the oscillation), and interpreted as differences in decoding regression coefficients (β), can either be a phase modulation or an amplitude modulation. When the difference is a phase modulation (top panel), the two stimuli can be distinguished as the black stimulus evokes an earlier response. As shown in the panels, β would have opposite signs between the two time points, which, as we will discuss, is relevant in interpreting decoding accuracy. When the difference is an amplitude modulation, two different possibilities exist. The first possibility (middle panel) is a decrease in power, such that β would again have opposite signs between the two marked time points. The second possibility (bottom panel) is an additive shift in the signal, which does not cause a sign inversion between the two time points. Therefore, when considered univariately, changes between stimuli in either the phase or amplitude of their evoked responses can be used for decoding.

Assuming for simplicity that all channels share the same kind of evoked response (for example having the same sign), Figure 2*B* illustrates two ways in which phase differences in evoked responses could manifest in decoding accuracy at the multi-channel level. The horizontal arrows reflect the phase difference between the two stimuli. In one type, the latency of the response is different between the stimuli, such that the phase modulation occurs earlier for some stimuli than for others; critically, this delay is global, that is, consistent across channels (Fig. 2*B*, left panel—where all arrows have the same length and orientation). In the other type, it is the relative differences in phase between channels that discriminates between stimuli (Fig. 2*B*, right panel—where arrows have different lengths and orientations), without necessarily being a global temporal shift that is common to all channels (i.e., the arrows could sum up to zero across channels).

These two different multivariate patterns may afford very different physiological interpretations. The first type of multivariate phase modulations would speak to systematic differences in the timing of information processing; for example, if images of animate beings take less time to process in the brain, then the cascade of stimulus processing would progress faster and that would bring about a change in the time course of the decoding accuracy. The second type of multivariate phase modulations would instead refer to differential phasic patterns between the image categories above and beyond their speed of processing, which could be related to detectable differences in the neural coding of the images. For some information about whether or not standard MVPA can distinguish between these two types of phase modulation, see the Supplementary Results (Supplementary Fig. 1).

### Relative Versus Global Phase Differences between Stimuli: Is it Just Processing Speed?

On this theoretical basis, we now ask in empirical data: what is the specific nature of the multivariate phase modulation that allows an effective discrimination between stimuli? Is it that the global latency of the response (i.e., common over all sensors) is different between experimental conditions (i.e., stimuli), such that phase-locking occurs a bit earlier for some stimuli than for others and the delay is approximately consistent across channels (*Case 1*, as in Fig. 2*B*, left). Or is it that the relative phase between channels is discriminant between experimental conditions (*Case 2*, as in Fig. 2*B*, right), without necessarily being a global all-channel temporal shift? To differentiate these two possibilities, we used a method that can account for the global differences in the timing of the evoked responses: the Temporally Unconstrained Decoding Analysis (TUDA) approach presented in Vidaurre, Myers, et al. (2019).

In brief, TUDA is a (Bayesian) generative model consisting of a number of decoding models (either classifiers or regressors) together with a data-driven estimation of when each decoding model should be used at each time-point within each trial, that is, the time courses of each decoding model (see section Materials and Methods). The critical feature of TUDA is that the temporal activation of each decoder can be different between trials. Hence, unlike standard decoding, TUDA can effectively accommodate between-trial temporal differences in the stimulus processing cascade. Crucially, these differences are global by definition. By modeling the global differences in the timing of the responses explicitly, TUDA can be used here to separate global from relative differences.

**Figure 3:**
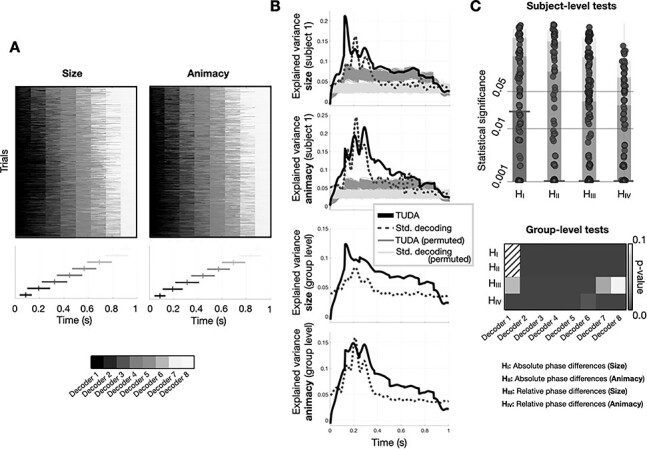
Phasic differences between stimuli in the oscillatory component can be due to 1) global differences in how fast the information is processed for each category, and 2) relative phasic differences between channels. (*A*) TUDA reveals between-trial temporal differences in stimulus processing; the matrices reflect the temporal occupancy of each decoding model for the two considered decoding problems. (*B*) Top panel: both TUDA and standard decoding exhibit significant decoding accuracy in discriminating animacy and size for a representative subject; statistical significance is given when the accuracy curve is higher than any of the (1000) permutations, represented in lighter colors. Bottom panels depict the average decoding accuracy across subjects. (*C*) Top: Manhattan plot showing *P*-values for the hypotheses of whether global latency is different between stimuli (size, H_I_; animacy, H_II_), and whether relative latency is different between image categories (size, H_III_; animacy, H_IV_); each dot corresponds to one statistical test for one subject, decoder and pair of category values (e.g., small vs. large); the colored boxes represents the area containing 95, 87.5, 75 and 50% (from lighter to darker) of the *P*-values, respectively, with a wider horizontal bar highlighting the median (50%). Bottom: group-level tests; note that H_I_ and H_II_ were not tested for the first decoder, since it always activates at the start of the trial.

For simplicity and computational efficiency, we trained the model on two supra-level categories into which the stimulus set could be grouped: the size of the stimulus (Size: small, medium, or large), and whether the image corresponds to an animate being (Animacy: yes or no). This way, we could estimate the parameters of the model using all the data for the 118 images and avoided the computational burden of inferring the model parameters for image pair. We set TUDA to use eight different decoders and applied it to the oscillatory component of the signal (see Fig. 1). Figure 3*A* shows, for one subject, the progression over the time within each trial of the decoders for Size and Animacy, where, for clarity, the trials were ordered according to the first principal component of the decoders’ activation time courses. At the bottom of the panel, the cross-trial average and standard deviation of each decoder’s timing of activation is shown. To verify the ability of TUDA and standard decoding to fit the data, we generated surrogated models to assess when TUDA and standard decoding reached a significant prediction, by simply permuting the labels of the stimuli and rerunning the models. As shown in Figure 3*B* for one subject (see Supplementary Fig. 2 for all subjects), both TUDA and standard decoding exhibited a larger accuracy than that of the surrogated data. For reference, the bottom panel of Figure 3*B* depicts the average explained variance across subjects. Note that TUDA had only 8 decoders as opposed to the 250 for standard decoding (i.e., one per time point).

TUDA provides a time-course of each decoding model, which captures when each decoding model should be used at each time-point within each trial. By construction, any global (common to all sensors) delays in phase locking that discriminate between stimuli (Case 1—Fig. 2*B*, left) will be captured by these trial-specific (thus, image-category-specific) decoding model time courses (Fig. 3*A*). To verify this, we used permutation testing (i.e., by permuting the labels across trials) to see if differences in when the decoding models start their activation is predictive of Size and Animacy (hypotheses H_I_ and H_II_ in Fig. 3*C*). This yielded one *P*-value per subject, decoding model and pair of image categories. Figure 3*C* confirms the existence of global phasic differences between the image categories (Case 1); see section Materials and Methods. Using the non-parametric combination algorithm (Vidaurre, Woolrich, et al. 2019b; see section Materials and Methods), the bottom of the panel shows an aggregation of tests into group-level *P*-values, with one *P*-value per state. The results are highly significant at the group level.

Once the global delays are accounted for by the decoding model time courses in TUDA, all that remains in the TUDA’s decoding models that is explanatory of the perceived stimulus is the relative phase differences between image categories (Case 2: Fig. 2*B*, left). Therefore, the fact that TUDA’s decoding models are able to discriminate successfully is evidence of the existence of “relative” phase differences. To further confirm the importance of relative differences between categories, we also asked whether phase is more consistent within image categories than it is across image categories “during” the time that each decoder is active. For example, for the time points when Decoder 2 is active, we asked if phase locking is significantly higher within category than between category. If the answer is positive, this would confirm the existence of differential phase patterns between the image categories above and beyond their speed of processing. To quantify phase locking, we used the Phase Locking Factor (PLF; Tallon-Baudry et al. 1997), which measures, for any given time point, how similar for each channel are the values of the phase across trials within a certain frequency band (see section Materials and Methods). We again used permutation testing to assess, for each decoder (i.e., for the time points where each decoder is active), whether PLF was higher within than between image categories. In this second type of test, Hypotheses H_III_ and H_IV_ correspond respectively to Size and Animacy, with one test per subject, decoder and pair of image categories. Figure 3*C* shows that more than half of the *P*-values are significant for both conditions, with a median *P*-value of <0.01 for Animacy. Again, the results are highly significant at the group level.

In summary, these results suggest the existence of significant phase differences between stimuli at two different levels, which speak to two different physiological mechanisms: global shifts in phase, attributed to the stimuli having diverse processing latencies; and relative phase differences, attributed to the existence of stimulus-specific, multivariate phase configurations. See the Supplementary Results for more information about the relation of these results to other concepts, such as spatially local-versus-global synchronization, inter-trial phase coherence, and inter-regional phase coupling.

### Decoding Accuracy Emerges from both Oscillatory and Non-Oscillatory Components

So far, we have shown that the phase of the oscillatory component can carry different types of information about the stimuli. We now consider the extent to which there is distinct and complementary information available between the oscillatory and the non-oscillatory components. Furthermore, we show how some of the most prominent phenomena commonly observed in decoding analysis can parsimoniously be explained by accounting for the separate contributions of these two components. To investigate this point, we used the temporal generalization matrix (TGM; see section Materials and Methods). The TGM extends conventional decoding that characterizes neural representations for each time point independently by elucidating how neural representations change or persist across time (King and Dehaene 2014). Given that this analysis is computationally faster than TUDA, we computed a TGM for each pair of images (i.e., 13 806 pairs), averaging across pairs.

**Figure 4:**
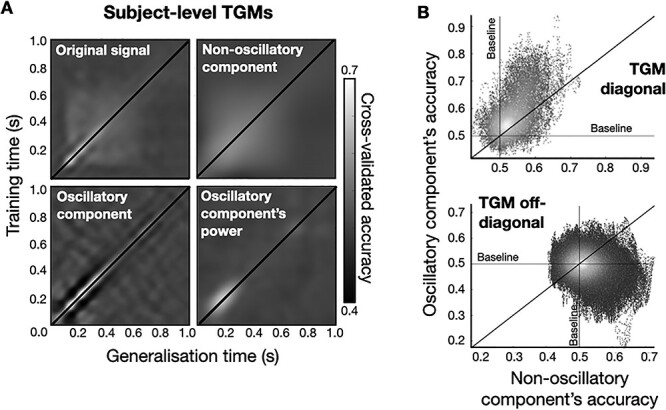
Non-oscillatory and oscillatory activity have a complementary contribution to MVPA accuracy. (*A*) TGMs for the original signal, for the non-oscillatory component, for the oscillatory component, and for the power of the oscillatory component. (*B*) TGM’s diagonal and TGM’s off-diagonal decoding accuracies of the non-oscillatory component versus oscillatory component. The oscillatory component dominates the diagonal and the non-oscillatory component dominates the off-diagonal of the TGM, indicating that the cross-generalization decoding accuracy is fundamentally based on the non-oscillatory component, whereas time point by time point decoding accuracy (i.e., the diagonal) is a mixture of both the non-oscillatory and the oscillatory components, but with a larger contribution from the oscillatory component.

Figure 4*A* shows (for one representative subject) the TGM for the original signal, the non-oscillatory component, the oscillatory component, and the power envelope of the oscillatory component (i.e., with no phase information). For ease of comparison, Supplementary Figure 3 shows, for each pair of images, the time-resolved decoding accuracy (i.e., the diagonal of the TGM) for one subject and on average; see Supplementary Figure 4 shows the group-average TGMs. The original signal’s decoding accuracy reflects a mixture from the decoding accuracy of the non-oscillatory and the oscillatory components: a linear regression model (done separately for each subject) can predict the original signal’s TGM from the non-oscillatory and the oscillatory component TGMs with 67% of explained variance on average across subjects. When using only the non-oscillatory, the explained variance was 9%; and when using only the oscillatory component, it was 59%. This suggests that both components are relevant for decoding in a complementary manner. Importantly, a model including the non-oscillatory component TGM, the oscillatory component TGM, and the power TGMs explains on average 7% more variance of the original signal TGM than a model that only includes non-oscillatory and power (*P* < 0.001 for all subject tests; permutation testing), which is a percentage decrement of 12%. This highlights the importance of phase in decoding analysis. Expanding on this, in Supplementary Results (Supplementary Fig. 5) we provide empirical evidence on the stimulus-specificity of phase-locking, as well as complementary decoding results on higher (>10 Hz) frequencies (Supplementary Fig. 6).

Figure 4*B* shows that the contribution the TGM’s diagonal is higher for the oscillatory component, but it does not generalize well over time. In contrast, the non-oscillatory component generalizes well over time, contributing more strongly to what is sometimes interpreted as the continuity in time of the neural representation. Specifically, the top panel of Figure 4*B* highlights that time points of high prediction accuracy in the TGM’s diagonal are supported by both the non-oscillatory and the oscillatory components, with a larger contribution of the oscillatory component (*P*-value < 0.001; permutation testing). The bottom panel suggests that cross-generalizing decoding accuracy (i.e., the accuracy that is away from the diagonal) is higher for the non-oscillatory component (*P*-value = 0.0182; permutation testing). Altogether, this analysis indicates that the non-oscillatory and the oscillatory component contribute to the overall TGM pattern in a complementary way.

**Figure 5:**
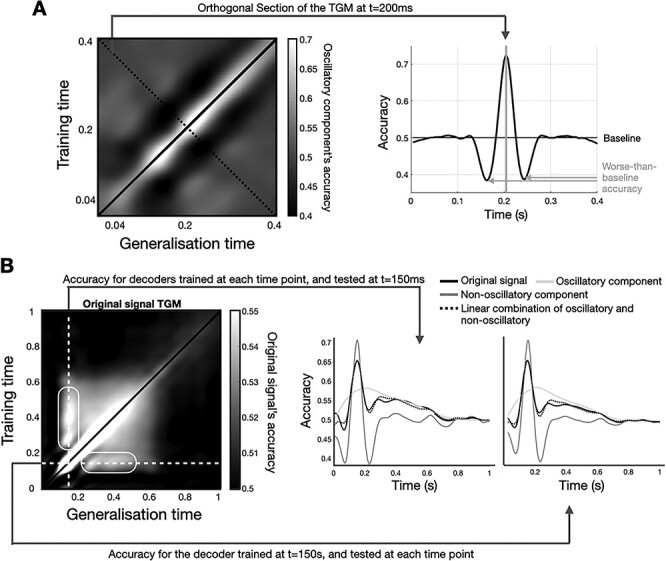
Interpretation of the TGM based on the separation between the oscillatory and non-oscillatory components. (*A*) The worse-than-average rebound relates to the oscillatory component. On the left, zoomed-in version of the oscillation-based TGM, within the 40–400 ms interval; on the right, a section of the TGM at ~200 ms. Result shown for one exemplary subject. (*B*) The stronger generalization at *t* = 150 ms is due to the combined effect of the non-oscillatory and oscillatory components (see main text). Result shown at the group level.

Other features of the TGM can be explained by considering the non-oscillatory and the oscillatory component separately. For example, a common pattern observed in decoding analysis is a worse-than-average rebound in decoding accuracy as we get away from the diagonal of the TGM (King and Dehaene 2014). Figure 5*A* depicts the oscillation-based TGM within the 0.04–0.4 s interval (left) for one example subject. The right panel represents a section of the matrix at a time point of approximately 200 ms and orthogonal to the timeline. Accuracy peaks at the diagonal and suffers a steep decrement below baseline at time points 25–75 ms away from the peak. This is consistent with the time occupied by half a cycle of a theta oscillation. We argue that this phenomenon may come about from the oscillatory component with no contribution from the non-oscillatory component. As demonstrated above schematically (top two panels of Fig. 2*A*), a rebound in decoding accuracy can come about through either a phase and/or an amplitude modulation causing a sign inversion in the differences in decoding regression coefficients between two time points. Therefore, certain patterns in the oscillatory component—but not in the non-oscillatory—can cause worse than baseline accuracy in the decoding.

Another common pattern observed in decoding analysis is a stronger generalization in regions of the TGM that stem, vertically and horizontally after brief period of depression, from the time points of maximum accuracy in the diagonal—as indicated (at the group level) as rounded boxes in the top left panel of Figure 5*B*. This phenomenon is sometimes interpreted as reactivation of a neuronal representation (King and Dehaene 2014). Instead, here we demonstrate that this phenomenon can parsimoniously be explained by an account of how decoding accuracy provided by the non-oscillatory component and the oscillatory-based phenomenon described in Figure 5*A* combine. In particular, we observe that 1) the initial depression of accuracy is caused by the peaks and troughs of the oscillatory component (Fig. 5*A*); and that 2) once such depression is passed, the reactivation is due to the fact that the non-oscillatory component’s accuracy takes longer to decay than it takes the oscillatory component’s accuracy to come back to baseline. This is shown quantitatively by regressing the non-oscillatory and oscillatory components’ accuracies onto the original signal’s accuracy, the prediction of which is shown as a dotted line in the right panels of Figure 5*B* (explained variance is 96.5 and 97.7%, respectively). Note that the non-oscillatory component’s generalized accuracy has its maximum later than 150 ms (i.e., it does better in subsequent time points than at the time point when it was trained); this is only due to the fact that this component’s relevance in the decoding occurs altogether later in the trial. Further note that this reactivation does not manifest in the non-oscillatory and oscillatory components alone (Fig. 4*A*), since it is the interaction of both which brings about this effect.

In summary, the key features of the TGM can be parsimoniously explained to a large extent when considering the oscillatory and non-oscillatory components separately, which should be taken into account when interpreting MVPA results.

## Discussion

In this paper, we have shown that two dissociable phase-related mechanisms can bring about decoding accuracy in MVPA on electrophysiological data: on the one hand, there are differences in the latency of processing, related to the possibility that some images are processed quicker than others; on the other hand, there are differential patterns of phase across channels, which can be attributed to the specifics of each neural representation. These two types of effects may speak to distinct neural phenomena affording very different cognitive interpretations. Importantly, their relative influence can determine how MVPA can be used in different scientific contexts. For example, when the timing of the response is uncertain as is the case with imagery (given the lack of an external trigger), MVPA likely needs to rely on relative phase differences given that global phase differences are difficult to control for. The fact that decoding has been applied in this setting successfully (Dijkstra et al. 2020; Xie et al. 2020) brings additional evidence of the existence of such relative phase differences in visual processing as measured by MEG. In cases where the spatial resolution of MEG might be insufficient to capture fine-grained relative differences, the global differences could be the only driver of a successful prediction. For example, this could be the case in pain processing, where a large part of the relevant signal originates from deep regions that are harder to access by non-invasive electrophysiological modalities (Tracey and Mantyh 2007). As shown in the Supplementary Results, an integrative approach that combines all channels, trials and time points in one model is an efficient and direct way of disambiguating these differences than time point by time point MVPA.

Furthermore, by breaking the signal into its oscillatory and non-oscillatory constituents, we found that the patterns of decoding accuracy in MVPA electrophysiological studies commonly observed in the literature can be well explained from the distinct contributions of these components, showing that the interpretation of these patterns may be misleading if we do not account for these distinct contributions. For example, certain patterns observed in the TGM are often interpreted as reactivation of mental representations, without considering that these patterns might arise from basic properties of the signal. While the cognitive interpretation is not necessarily incorrect, we suggest that more robust interpretations will emerge from embracing the complexity of the signal and dissecting how its fundamental properties relate to cognition. Although not investigated here (since TUDA was run here only on the oscillatory component), TUDA might also exhibit caveats when the different signal components are presented in a conflated manner. These analyses also stressed the importance of multivariate over purely univariate analyses, supporting the claim that the former are better able to account for systemic nature of the nervous system (Kragel et al. 2018).

We note that the scope of our conclusions covers MVPA applied in a time-resolved manner (time point by time point) on electrophysiological data, but not necessarily on neuroimaging data. We also note that the analysis performed here was by no means intended to be exhaustive in accounting for all the different features contained in the data. For simplicity, we removed all information content above 10 Hz, thereby disregarding higher frequencies such as those in the gamma band, which has purportedly a critical role in cognition (Herrmann et al. 2004). Nor did we look at the how the interaction between the non-oscillatory and the oscillatory components can predict the perceived stimulus. Importantly, our conclusions stand regardless of the fact that other aspects in the signal (such as >10-Hz frequencies) also carry information about the stimulus. Future investigations will be needed to understand the role of these other components. As in all analyses, there are to some extent arbitrary choices made in our analyses. This includes the choice of decoder, choosing 8 decoders in TUDA, or performing the decomposition of the signal into oscillatory and non-oscillatory components using local regression. In future applications it may be beneficial to optimize some of these choices, for example, using cross-validation. Although we did not do this here, the default modeling choices we used were, however, able to describe the data sufficiently well in order to answer our specific questions.

An important open question about the mechanistic underpinnings of stimulus processing is whether the stimulus-specific evoked activity is caused by a reorganization of the ongoing phasic trajectories (i.e., through phase resetting) or is otherwise generated independently of the ongoing phase. Whereas we showed here that there is information contained in the patterns of phase-locking across trials, this does not necessarily imply the existence of phase-resetting (Shah et al. 2004; Mäkinen et al. 2005; Mazaheri and Jensen 2006). Distinguishing between these two cases is not obvious in non-invasive electrophysiological data (Sauseng et al. 2007), and, at the bare minimum, requires a sufficient baseline period that we lack here. We however speculate that, most likely, the observed effects will not be purely caused by neither phase-resetting nor a separate event-related activation, but instead be some mixture of both, with proportions that depend on the brain region and the type of stimulus (Fell et al. 2004). It is also possible that phase-resetting occurs, but studies focused on univariate analysis could not find it, simply because the effect relies on subtle differences across channels that can only be identified using a multivariate approach.

More broadly, there is the more general question of how the whole-brain ongoing (pre-stimulus) phase configuration of the brain in slow frequencies influences stimulus processing at higher frequencies. Previously, we showed that there exist reliable whole-brain patterns of phase coherence in theta and alpha at rest (Vidaurre, Hunt, et al. 2018), and that these configurations hold some relation to gamma frequency stimulus responses (Hirschmann et al. 2019). Considering these and other many studies about the influence of the low frequency ranges on diverse aspects of cognition (Monto et al. 2008; He 2014; Baria et al. 2017) and the different ways that ongoing brain states modulate neural responses (Podvalny et al. 2019; McCormick et al. 2020), it is clear that part of the large variability observed in the neural responses could be explained by the ongoing, large-scale network activity and concurrent phase trajectories, and that accounting by these factors could arguably improve the capacity of MVPA to produce successful predictions.

## Conclusion

In the last decade, brain decoding models have emerged as powerful tools to predict mental constructs from electrophysiological measurements of human brains. The fact that these models are able to obtain significant predictions from non-invasive data suggests that macroscale brain activity reflects aspects of stimulus-specific computations at some level in the brain. However, the power in prediction has come at the expense of explanation: the statistical methods employed leave open what it is about the data that allows the successful prediction, and thus miss crucial information for theory building. Here, we propose that macroscale brain signals contain stimulus-specific information at various different levels: in the differences of the latency of response between image categories; in relative difference of phase between channels; and in the non-oscillatory, stable component of the signal. We argue that in dissociating these components and understanding their potential hierarchical organization may open new routes to understand the multifaceted aspects of information processing in the brain.

## Funding

Novo Nordisk Emerging Investigator Award (NNF19OC-0054895 to D.V.); European Research Council (ERC-StG-2019-850404 to D.V.); German Research Council (DFG) (CI241/1-1, CI241/3-1 to R.C.); European Research Council (ERC-StG-2018-803370 to R.C.); NIHR Oxford Health Biomedical Research Centre, by the Wellcome Trust (106183/Z/14/Z to M.W.W.); MRC UK MEG Partnership Grant (MR/K005464/1).

## Notes

We thank Nicolás Gravel for useful comments and technical help. *Conflict of Interest*: The authors declare no conflicts of interest.

## Supplementary Material

Supplementary_bhab189

## References

[ref1] Baria AT, Maniscalco B, He BJ. 2017. Initial-state-dependent, robust, transient neural dynamics encode conscious visual perception. PLoS Comput Biol. 13:e1005806. 29176808 10.1371/journal.pcbi.1005806 PMC5720802

[ref2] Bullock TH, McClune MC, Enright JT. 2003. Are the electroencephalograms mainly rhythmic? Assessment of periodicity in wide-band time series. Neuroscience. 121:233–252. 12946714 10.1016/s0306-4522(03)00208-2

[ref3] Buzsáki G, Draguhn A. 2004. Neuronal oscillations in cortical networks (2004). Science. 25:1926–1929. 10.1126/science.1099745 15218136

[ref4] Buzsáki G, Logothetis N, Singer W. 2013. Scaling brain size, keeping timing: evolutionary preservation of brain rhythms. Neuron. 80:751–764. 24183025 10.1016/j.neuron.2013.10.002 PMC4009705

[ref5] Buzsáki G, Lopez da Silva FH. 2012. High frequency oscillations in the intact brain. Prog Neurobiol. 98:241–249. 22449727 10.1016/j.pneurobio.2012.02.004 PMC4895831

[ref6] Canolty RT, Knight RT. 2010. The functional role of cross-frequency coupling. Trends Cogn Sci. 14:506–515. 20932795 10.1016/j.tics.2010.09.001 PMC3359652

[ref6a] Carlson TA, T Grootswagers T, Robinson AK. 2019. An introduction to time-resolved decoding analysis for M/EEG. arXiv preprint arXiv:1905.04820.

[ref7] Cichy RM, Pantazis D, Oliva A. 2014. Resolving human object recognition in space and time. Nat Neurosci. 17:455–462. 24464044 10.1038/nn.3635 PMC4261693

[ref8] Cichy RM, Pantazis D, Oliva A. 2016. Similarity-based fusion of MEG and fMRI reveals spatio-temporal dynamics in human cortex during visual object recognition. Cereb Cortex. 26:3563–3579. 27235099 10.1093/cercor/bhw135 PMC4961022

[ref9] Clarke A, Devereux BJ, Tyler LK. 2018. Oscillatory dynamics of perceptual to conceptual transformations in the ventral visual pathway. J Cogn Neurosci. 30:1590–1605. 30125217 10.1162/jocn_a_01325

[ref10] Clarke A, Taylor KI, Devereux B, Randall B, Tyler LK. 2013. From perception to conception: how meaningful objects are processed over time. Cereb Cortex. 23:187–197. 22275484 10.1093/cercor/bhs002 PMC3619663

[ref11] Dijkstra N, Ambrogioni L, Vidaurre D, van Gerven M (2020). Neural dynamics of perceptual inference and its reversal during imagery. Elife 9, e53588. 32686645 10.7554/eLife.53588 PMC7371419

[ref12] Donoghue T, Haller M, Peterson EJ, Varma P, Sebastian P, Gao R, Noto T, Lara AH, Wallis JD, Knight RT et al. 2020. Parameterizing neural power spectra into periodic and aperiodic components. Nat Neurosci. 23:1655–1665. 33230329 10.1038/s41593-020-00744-x PMC8106550

[ref13] Fell J, Dietl T, Grunwald T, Kurthen M, Klaver P, Trautner P, Schaller C, Elger CE, Fernández G. 2004. Neural bases of cognitive ERPs: more than phase reset. J Cogn Neurosci. 16:1595–1604. 15601521 10.1162/0898929042568514

[ref14] Friedman J, Hastie T, Tibshirani R. 2001. The elements of statistical learning: data mining, inference, and prediction. New York: Springer.

[ref15] Grootswagers T, Wardle SG, Carlson TA. 2017. Decoding dynamic brain patterns from evoked responses: a tutorial on multivariate pattern analysis applied to time series neuroimaging data. J Cogn Neurosci. 29:677–697. 27779910 10.1162/jocn_a_01068

[ref16] Jensen O, Colgin LL. 2007. Cross-frequency coupling between neuronal oscillations. Trends Cogn Sci. 18:267–269. 10.1016/j.tics.2007.05.003 17548233

[ref17] Jensen O, Gips B, Bergmann TO, Bonnefond M. 2014. Temporal coding organized by coupled alpha and gamma oscillations prioritize visual processing. Trends Neurosci. 37:357–369. 24836381 10.1016/j.tins.2014.04.001

[ref18] Jones SR . 2016. When brain rhythms aren’t “rhythmic”: implication for their mechanisms and meaning. Curr Opin Neurobiol. 40:72–80. 27400290 10.1016/j.conb.2016.06.010 PMC5056821

[ref20] Haynes JD, Rees G. 2006. Decoding mental states from brain activity in humans. Nat Rev Neurosci. 7:523–534. 16791142 10.1038/nrn1931

[ref21] He BJ . 2014. Scale-free brain activity: past, present and future. Trends Cogn Sci. 18:480–487. 24788139 10.1016/j.tics.2014.04.003 PMC4149861

[ref22] Herrmann CS, Munk MHJ, Engel AK. 2004. Cognitive functions of gamma-band activity: memory match and utilization. Trends Cogn Neurosci. 8:347–355. 10.1016/j.tics.2004.06.006 15335461

[ref23] Hirschmann J, Baillet S, Woolrich MW, Schnitzler A, Vidaurre D, Florin E. 2019. Spontaneous network activity <35 Hz accounts for variability in stimulus-induced gamma responses. Neuroimage. 207:116374. 31759115 10.1016/j.neuroimage.2019.116374 PMC8111242

[ref24] Huang NE, Wu Z, Long SR, Arnold KC, Chen X, Blank K. 2009. On instantaneous frequency. Adv Adapt Data Anal. 1:177–229.

[ref25] Kayser C, Ince RAA, Panzeri S. 2012. Analysis of slow (theta) oscillations as a potential temporal reference frame for information coding in sensory cortices. PLoS Comput Biol. 8:e1002717. 23071429 10.1371/journal.pcbi.1002717 PMC3469413

[ref26] King JR, Dehaene S. 2014. Characterizing the dynamics of mental representations: the temporal generalization method. Trends Cogn Neurosci. 18:203–210. 10.1016/j.tics.2014.01.002 PMC5635958 24593982

[ref27] Kragel PA, Koban L, Feldman Barrett LF, Wager TD. 2018. Representation, pattern information, and brain signatures: from neurons to neuroimaging. Neuron. 99:257–273. 30048614 10.1016/j.neuron.2018.06.009 PMC6296466

[ref28] Linkenkaer-Hansen K, Nikouline VV, Palva J, Ilmoniemi RJ. 2001. Long-range temporal correlations and scaling behavior in human brain oscillations. J Neurosci. 21:1370–1377. 11160408 10.1523/JNEUROSCI.21-04-01370.2001 PMC6762238

[ref29] Lundqvist M, Rose J, Herman P, Brincat SL, Buschman TJ, Miller EK. 2016. Gamma and Beta bursts underlie working memory. Neuron. 90:152–164. 26996084 10.1016/j.neuron.2016.02.028 PMC5220584

[ref30] Mäkinen V, Tiitinen H, May P. 2005. Auditory event-related responses are generated independently of ongoing brain activity. Neuroimage. 24:961–968. 15670673 10.1016/j.neuroimage.2004.10.020

[ref31] Mazaheri A, Jensen O. 2006. Posterior alpha activity is not phase-reset by visual stimuli. PNAS. 103:2948–2952. 16473952 10.1073/pnas.0505785103 PMC1413767

[ref32] Melloni L, Carlos Molina C, Marcela Pena C, Torres D, Singer W, Rodriguez E. 2007. Synchronization of neural activity across cortical areas correlates with conscious perception. J Neurosci. 27:2858–2865. 17360907 10.1523/JNEUROSCI.4623-06.2007 PMC6672558

[ref33] Monto S, Palva S, Voipio J, Palva JM. 2008. Very slow EEG fluctuations predict the dynamics of stimulus detection and oscillation amplitudes in humans. J Neurosci. 28:8268–8272. 18701689 10.1523/JNEUROSCI.1910-08.2008 PMC6670577

[ref34] McCormick DA, Nestvogel DB, He BJ. 2020. Neuromodulation of brain state and behaviour. Annu Rev Neurosci. 43:391–415. 32250724 10.1146/annurev-neuro-100219-105424 PMC12237593

[ref37] Pesarin F, Salmaso L. 2010. Permutation tests for complex data: theory, applications and software. West Sussex, England, UK: John Wiley and Sons.

[ref38] Pfurtscheller G, Lopes da Silva FH. 1999. Event-related EEG/MEG synchronization and desynchronization: basic principles. Clin Neurophysiol. 110:1842–1857. 10576479 10.1016/s1388-2457(99)00141-8

[ref39] Podvalny E, Flounders MW, King LE, Holroyd T, He BJ. 2019. A dual role of prestimulus spontaneous neural activity in visual object recognition. Nat Commun. 10:3910. 31477706 10.1038/s41467-019-11877-4 PMC6718405

[ref40] Quinn AJ, Lopes-dos-Santos V, Huang N, Liang WK, Juan CH, Yeh JR, Nobre AC, Dupret D, Woolrich MW. 2021. Within-cycle instantaneous frequency profiles report oscillatory waveform dynamics. BioRxiv preprint. 10.1101/2021.04.12.439547. PMC7611760 34406888

[ref41] Rodriguez E, George N, Lachaux JP, Martinerie J, Renault B, Varela FJ. 1999. Perception's shadow: long-distance synchronization of human brain activity. Nature. 397:430–433. 9989408 10.1038/17120

[ref42] Sauseng P, Klimesch W. 2008. What does phase information of oscillatory brain activity tell us about cognitive processes? Neurosci Biobehav Rev. 32:1001–1013. 18499256 10.1016/j.neubiorev.2008.03.014

[ref43] Sauseng P, Klimesch W, Gruber WR, Hanslmayr S, Freunberger R, Doppelmayr M. 2007. Are phase-related potential components generated by phase resetting of brain oscillations? A critical discussion. Neuroscience. 146:1435–1444. 17459593 10.1016/j.neuroscience.2007.03.014

[ref44a] Shah AS, Bressler SL, Knuth KH, Ding M, Mehta AD, Ulbert I, Schroeder CE. 2004. Neural dynamics and the fundamental mechanisms of event-related brain potentials. Cereb Cortex. 14:476–483. 15054063 10.1093/cercor/bhh009

[ref44] Steriade M . 2001. Impact of network activities on neuronal properties in corticothalamic systems. J Neurophysiol. 86:1–39. 11431485 10.1152/jn.2001.86.1.1

[ref45] Takacs A, Mückschel M, Roessner V, Beste C. 2020. Decoding stimulus-response representations and their stability using EEG-based multivariate pattern analysis. Cerebl Cortex Commun. 1:tgaa016. 10.1093/texcom/tgaa016 PMC8152870 34296094

[ref46] Tallon-Baudry C, Bertrand O, Delpuech C, Pernier J. 1997. Oscillatory γ-band (30–70 Hz) activity induced by a visual search task in humans. J Neurosci. 17:722–734. 8987794 10.1523/JNEUROSCI.17-02-00722.1997 PMC6573221

[ref47] Tong F, Pratte MS. 2012. Decoding patterns of human brain activity. Annu Rev Psychol. 63:483–509. 21943172 10.1146/annurev-psych-120710-100412 PMC7869795

[ref48] Tracey I, Mantyh PW. 2007. The cerebral signature for pain perception and its modulation. Neuron. 55:377–391. 17678852 10.1016/j.neuron.2007.07.012

[ref49] van Ede F, Quinn AJ, Woolrich MW, Nobre AC. 2018. Neural oscillations: sustained rhythms or transient burst-events? Trends Neurosci. 41:415–417. 29739627 10.1016/j.tins.2018.04.004 PMC6024376

[ref50] Vidaurre D, Hunt LT, Quinn AJ, Hunt BSE, Brookes MJ, Nobre AC, Woolrich MW. 2018. Spontaneous cortical activity transiently organises into frequency specific phase-coupling networks. Nat Commun. 9:2987. 30061566 10.1038/s41467-018-05316-z PMC6065434

[ref51] Vidaurre D, Abeysuriya R, Becker R, Quinn AJ, Alfaro-Almagro F, Smith SM, Woolrich MW. 2018b. Discovering dynamic brain networks from Big Data in rest and task. Neuroimage. 180:646–656. 28669905 10.1016/j.neuroimage.2017.06.077 PMC6138951

[ref52] Vidaurre D, Quinn AJ, Dupret D, Tejero-Cantero A, Woolrich MW. 2016. Spectrally resolved fast transient brain states in electrophysiological data. Neuroimage. 126:81–95. 26631815 10.1016/j.neuroimage.2015.11.047 PMC4739513

[ref53] Vidaurre D, Myers NE, Stokes M, Nobre AC, Woolrich MW. 2019. Temporally unconstrained decoding reveals consistent but time-varying stages of stimulus processing. Cereb Cortex. 29:863–874. 30535141 10.1093/cercor/bhy290 PMC6319313

[ref54] Vidaurre D, Woolrich MW, Winkler AM, Karapanagiotidis T, Smallwood J, Nichols TE. 2019b. Stable between-subject statistical inference from unstable within-subject functional connectivity estimates. Hum Brain Mapp. 40:1234–1243. 30357995 10.1002/hbm.24442 PMC6492297

[ref55] Weichwald S, Meye T, Özdenizci O, Schölkopf B, Ball T, Grosse-Wentrup M. 2015. Causal interpretation rules for encoding and decoding models in neuroimaging. Neuroimage. 110:48–59. 25623501 10.1016/j.neuroimage.2015.01.036

[ref56] Xie S, Kaiser D, Cichy RM. 2020. Visual imagery and perception share neural representations in the alpha frequency band. Curr Biol. 30:1–7. 32750335 10.1016/j.cub.2020.07.023 PMC7416104

