# Supplementary material for: Dissociable Components of Information Encoding in Human Perception
Source: Cereb Cortex. 2021 Jul 22;31(12):5664–75. doi: 10.1093/cercor/bhab189 (PMC8568005; doi:10.1093/cercor/bhab189)
Supplement: Supplementary_bhab189 [file supplementary_bhab189.docx]

**Supplementary Methods**

**Measuring channel relevance for decoding**

In order to get an estimation of how important is a channel for the decoding, we can compute, for each channel *j*, time point *t* and pair of stimuli *g* and *h*, an unsigned t-statistic representing how important is that channel for the decoding at *t*:

$b_{tj}^{(gh)}=c \left| \frac{\mu_{tgj}- \mu_{thj}}{\sqrt{s_{tgj}^{2}+}s_{tgj}^{2}} \right|$,

where $\mu_{tgj}$ and $s_{tgj}^{2}$ are, respectively, the mean and the variance of channel *j* at time point *t* for stimulus *j*, and *c* is a constant. We then aggregated these for each stimulus, with respect to all the other stimuli, such as:

$\bar{b}_{tj}^{g}=\frac{1}{p-1} \sum_{h\neq g} b_{tj}^{(gh)},$ where *p* is the number of stimuli.

These $\bar{b}_{tj}^{g}$ values represent how relevant is channel *j* at time *t* to discriminate stimulus *g*. These were used instead of multivariate decoding weights because of their higher interpretability (Haufe et al, 2014).

**Supplementary Results**

**Can we disambiguate absolute-vs-relative phase differences with standard decoding?**

In this paper, we have used TUDA to investigate the distinction between absolute-vs-relative phase differences. Since TUDA es mathematically and computationally more complex than standard decoding (where we perform decoding for each time point of the trial separately, while ignoring between-trial differences), it is useful to ask whether we can simply use the latter to disentangle the two types of phase differences. To investigate this, we generated synthetic data for two conditions (blue and red) where an additive sinusoidal signal was used to represent the processing of the stimulus, similarly to **Fig 2B**. The differences between the two conditions or stimuli are given purely by phase, i.e. by when exactly the sinusoid has been injected. As illustrated in **Fig 2B**, this could be done such that the differences were relative of absolute (plus some random jitter). Within the absolute differences, we make one further distinction: whether or not the modulation has the same sign for all channels. We adjusted the parameters of the simulations so that the total amount of signal difference between the conditions is approximately equivalent for the three cases. **Fig SI-1A** shows an example of each type of modulation for one trial per condition (per condition), where each line corresponds to one channel. **Fig SI-1B**, shows that, by looking at prediction accuracy, standard decoding is in general unable to disambiguate between the three cases. **Fig SI-1C** shows that it is however possible to disentangle absolute-vs-relative at single time points by looking at the mean of the decoding coefficients across sensors per time point —but only if the ERP/Fs have the same polarity across channels. This is particularly untrue when working in source space due to the sign ambiguity of the source reconstruction process. In summary, while it is still possible to disambiguate relative vs absolute phase differences using standard MVPA in some cases, a more integrative approach (such as TUDA) can be more efficient and generally applicable for this purpose.

**Absolute-vs-relative phase differences: relation to other concepts**

We have discussed two types of stimulus-specific phase differences: global and relative. This distinction is related, but different, to other classifications already analysed in the literature. One is the separation between spatially global and local phase synchronisation (Rodríguez et al., 1999; Sauseng and Klimesch, 2008), where global/local is used within a spatial context. In theory, global differences can occur within a subset of the channels, regardless of whether these channels are spatially local to each other or spanning wide extensions of the brain. Similarly, the fact that we found relative differences is unrelated to how these differences spatially distribute across the brain. Nevertheless, we would expect that global differences (in the sense used here) —potentially marked by the salience of the stimulus— would span more widely across the brain, whereas relative differences not necessarily so; for example in the case of subconscious perception (Melloni et al., 2017).

Another related pair of concepts is inter-trial coherence (ITC), measured for example by

the phase-locking factor (PLF; Tallon-Baudry et al., 1997); and inter-regional coupling (IRC), measured by the phase-locking vector (PLV; Lachaux et al., 1999). ITC and IRC describe the presence of phase locking between trials and channels respectively. The measures of global and relative shifts in phase that we are pointing at here are related to ITC and IRC, in the sense that they depend on the presence of ITC and IRC. However, they are otherwise distinct properties that describe how the phase changes *between different conditions* across different channels. Consider the two scenarios in **Fig 2B** (left vs right): in both, ITC occurs separately in all 5 channels across both conditions, giving rise to the observed non-zero trial-averaged responses. Importantly, thanks to the presence of ITC in all channels/conditions, IRC is also present in both scenarios, i.e. between any pair of channels for both condition. While IRC and ITC is present in both scenarios, only in the left case there is a global phase shift, and only in the right case there is a relative phase shift. Critically, only the latter scenario will produce a differential effect in IRC *between the conditions*, because shifting all phases at once for one of the two condition would leave the IRC unmodified at the trial level. In other words, while ITC and IRC are needed for these differences to exist, the distinction between global and relative shifts between conditions brings additional information that is not completely captured by ITC or IRC.

**Phase-locking is stimulus-specific**

We have shown that phase of the oscillatory component contains relevant information about the stimuli, and that MVPA can leverage this information to discriminate between the different image classes. We here provide further evidence that this is the case by showing that, not only that there (obviously) must exist at least a certain amount phase-locking for decoding analysis to succeed, but also that the nature of such phase-locking is stimulus-specific and thus is important to decoding.

Phase-locking is necessary because if the phases were completely random across trials, then trials with a negative field would cancel trials with a positive field, with the result that phase-based decoding would be impossible. Phase locking between trials at each time point can be quantified using the Phase Locking Factor (PLF; Tallon-Baudry et al., 1997), which measures, for any given time point, how similar for each channel are the values of the phase across trials within a certain frequency band (see Methods). To confirm this, **Fig SI-5A** shows, for one representative subject, the PLF per time point (averaged across channels) together with the time-resolved decoding accuracy (i.e. the diagonal of the TGM); the correlation of these two metrics is 0.94 (0.935 on average across subjects), highlighting the importance of phase-locking for MVPA. Furthermore, we computed a t-statistic $\bar{b}_{tj}^{g}$ representing how important a given channel *j* is for the discrimination of stimulus *g* at time point *t* (see Methods). More precisely, the “decoding weights” in the figure are univariate t-statistics instead of multivariate regression coefficients, because univariate statistics are more representative of the relevance of a feature than multivariate regression coefficients (Haufe et al 2014). Focusing on the peak of the phase-locking for an exemplary subject (*t*=200ms), **Fig SI-5B** confirms that the channels exhibiting a higher degree of phase coupling are also those that contribute more strongly to the decoding estimation; the scatter plot shows this relationship such that each point corresponds to a particular channel and stimulus. The histogram in **Fig SI-5C** shows the distribution of correlations between the PLF values and the decoding weights across channels, pooled across stimuli and subjects. The correlations are significantly higher than 0.0 (p<0.001, permutation testing) for all subjects.

Having shown that phase-locking is necessary, we further demonstrate that it is also stimulus-specific. Using permutation testing and focusing on *t*=0.2s, we tested whether there is more phase-locking between trials that correspond to the same stimulus than between trials that correspond to different stimuli (see Methods). **Fig SI-5C** presents the p-values across subjects and pairs of stimuli, showing that the timing and nature of such phase-locking is highly stimulus-specific (i.e. most p-values are significant).

**High-frequency TGMs**

In this work, we have separated the non-oscillatory part of the signal and an oscillatory component. For simplicity, we focused on the theta oscillation, even when higher frequencies are well known to be involved in visual processing. This decision was motivated by our interest of examining single elements of the signal one at a time, therefore avoiding more complex oscillatory patterns such as nested oscillations. For completeness, we also ran the standard decoding analysis (i.e. the TGM) for remaining part of the signal, i.e. containing frequencies higher than 10Hz. **Fig SI-6** shows the corresponding TGMs, for the same subject that we analysed before in **Fig 4** and **5** as well as at the group level. We can observe similar patterns to those observed in **Fig 4** and **5**, but occurring more shortly (i.e. faster) and with lower decoding accuracy. This suggests that the general points made in **Fig 4** and **5** also apply to higher frequencies.

**References**

Haufe S, Meinecke F, Görgen K, Dähne S, Haynes JD, Blankertz B and Biebmann F (2014). On the interpretation of weight vectors of linear models in multivariate neuroimaging. *NeuroImage* **87**, 96-110.

Lachaux JF, E. Rodriguez, J. Martinerie and F.J. Varela (1999). Measuring phase synchrony in brain signals. *Human Brain Mapping* **8**, 194-208.

**Supplementary Figures**


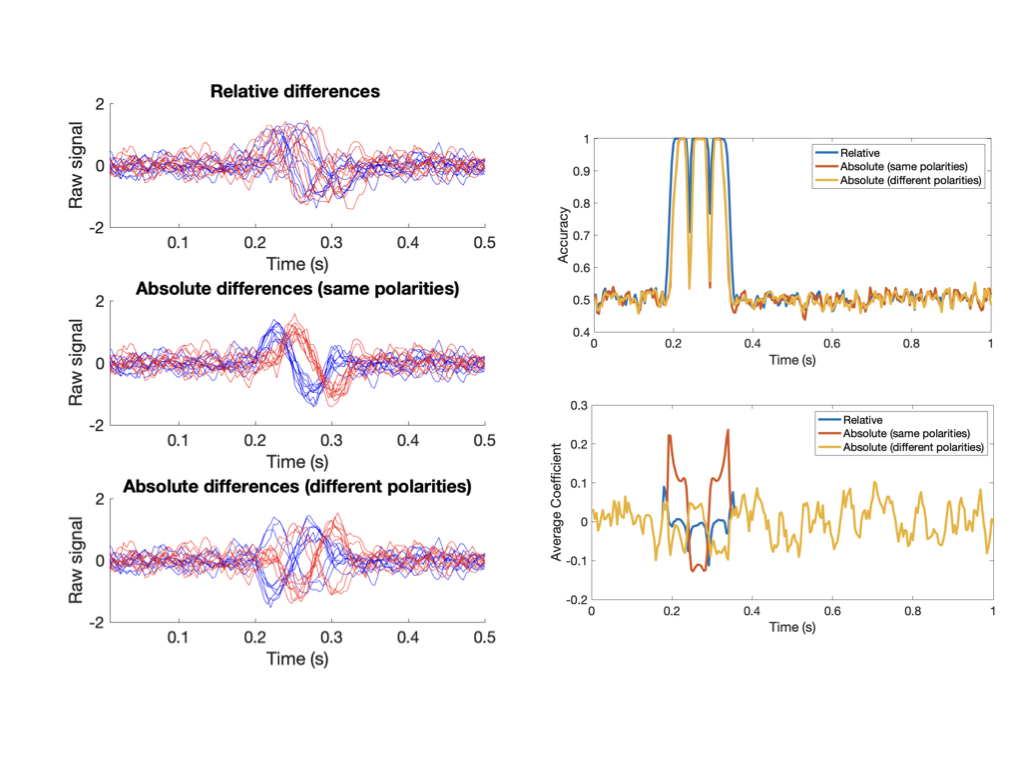


**Fig SI-1**. The distinction between absolute and relative phase differences can only be identified at the single time point level in specific cases. **A**: Synthetic data showing relative (top) and absolute differences (middle, where the ERP/F have the same polarities; bottom when the polarities are random). Each line represents one channel, and the two colours refer to two different stimuli; data is presented for one trial per stimuli. **B**: We applied standard decoding per time point. Standard decoding accuracy (time point by time point) is shown, which cannot disambiguate absolute and relative phase differences. **C**: The cross-channel average of the decoding coefficient can discriminate absolute and relative phase differences —but only when the polarity of the signal in the is consistent across channels.

**
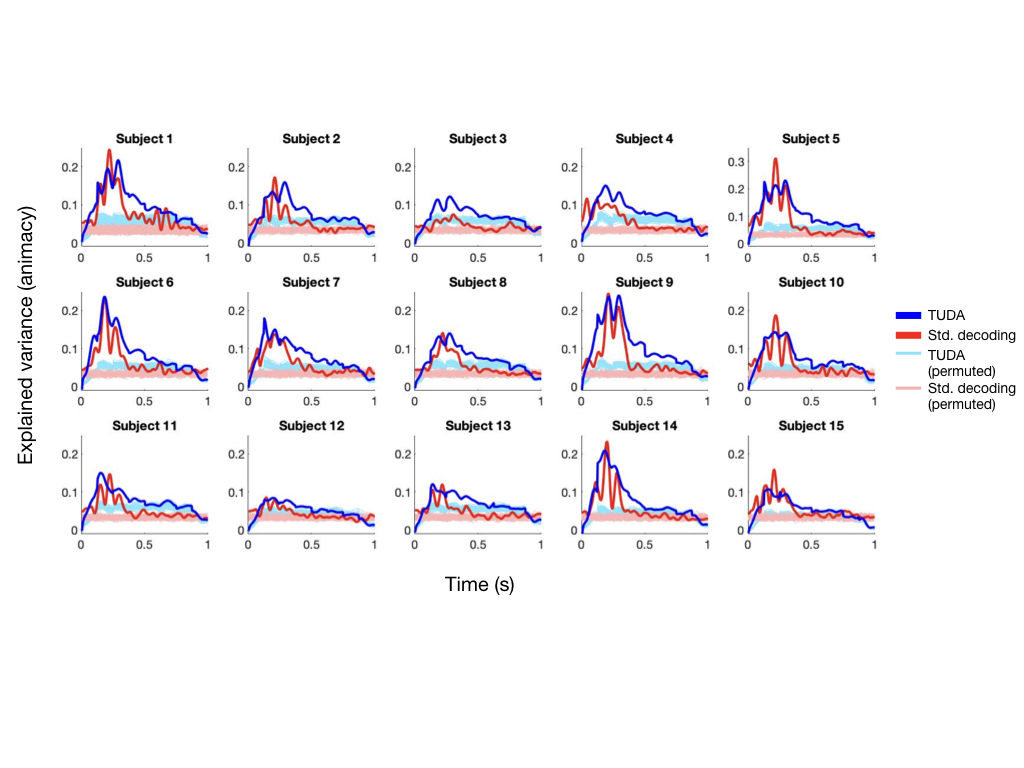
**

**
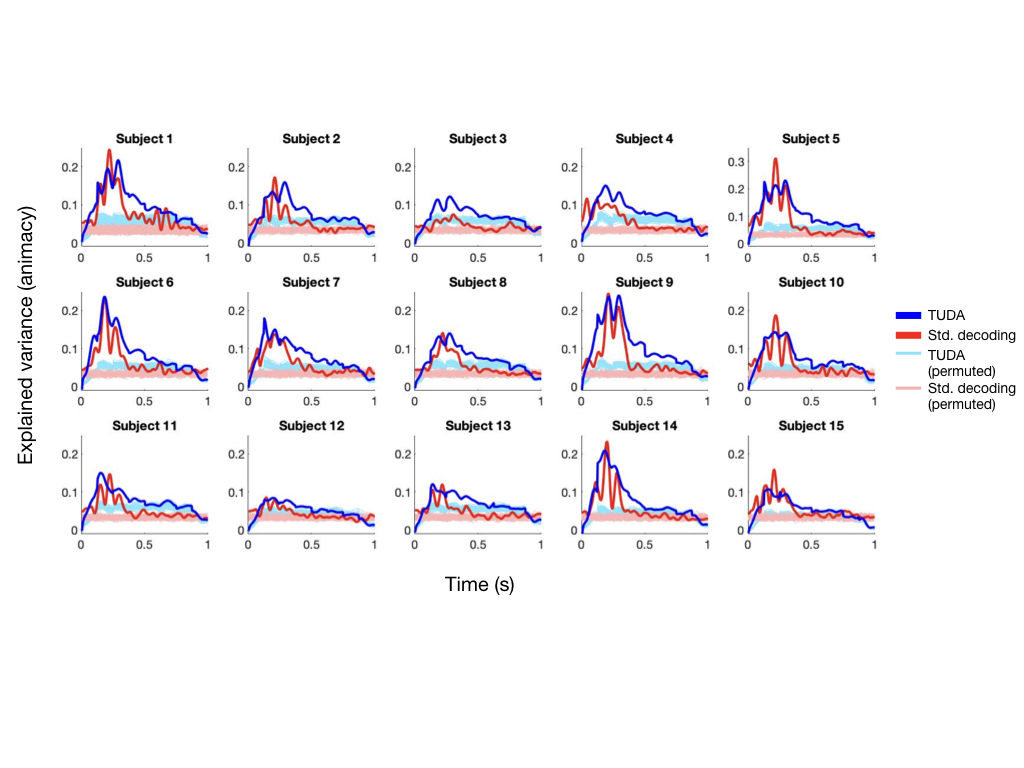
**

**Fig SI-2.** Subject by subject cross-validated decoding of size (top) and animacy (bottom), together with the decoding accuracy for 100 surrogates per subject (obtained by permuting the condition label).

**
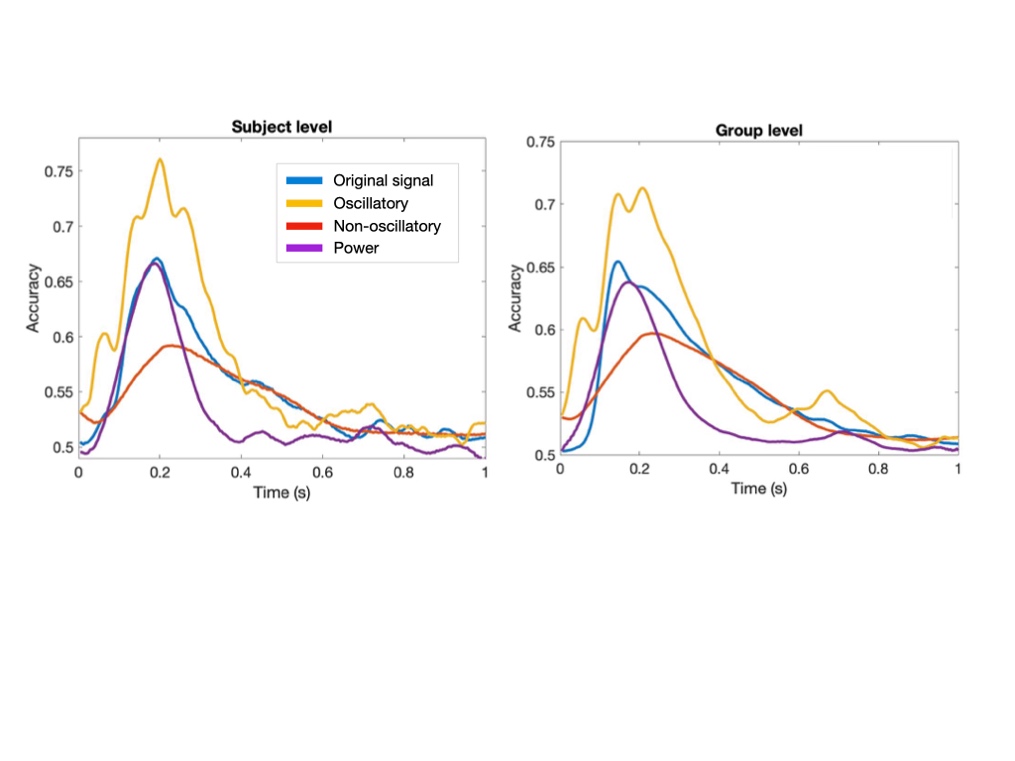
**

**Fig SI-3.** Time-resolved decoding accuracy for one subject and at the group level, averaged across pairs of images.


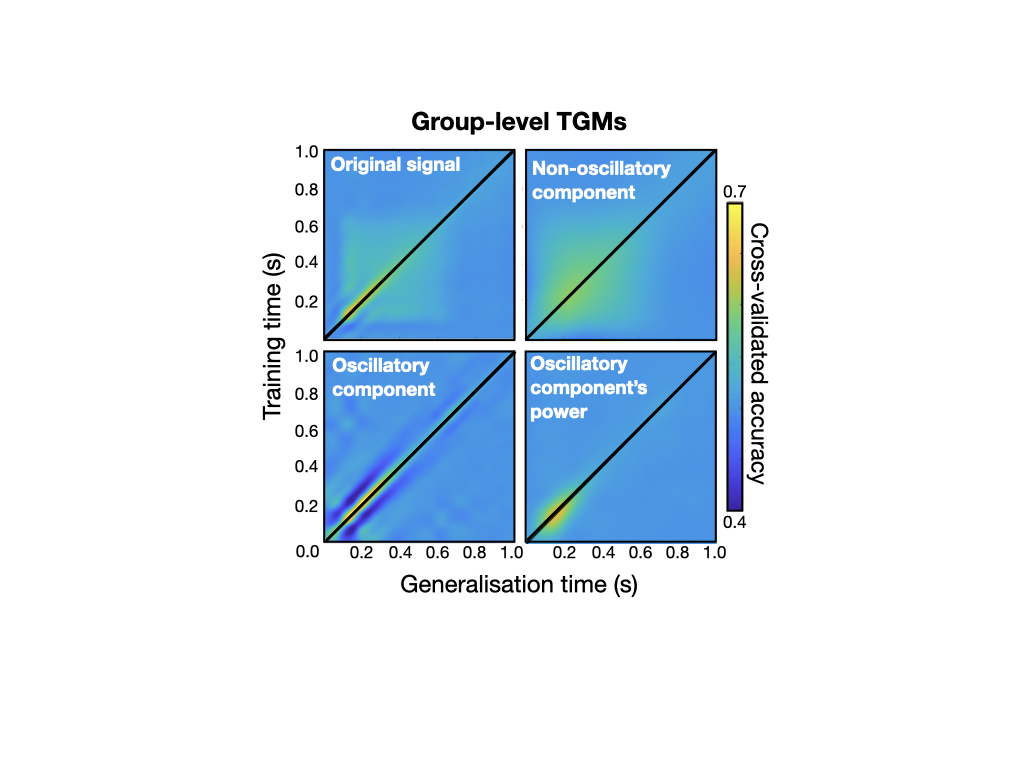


**Fig SI-4.** Group-level temporal generalisation matrices


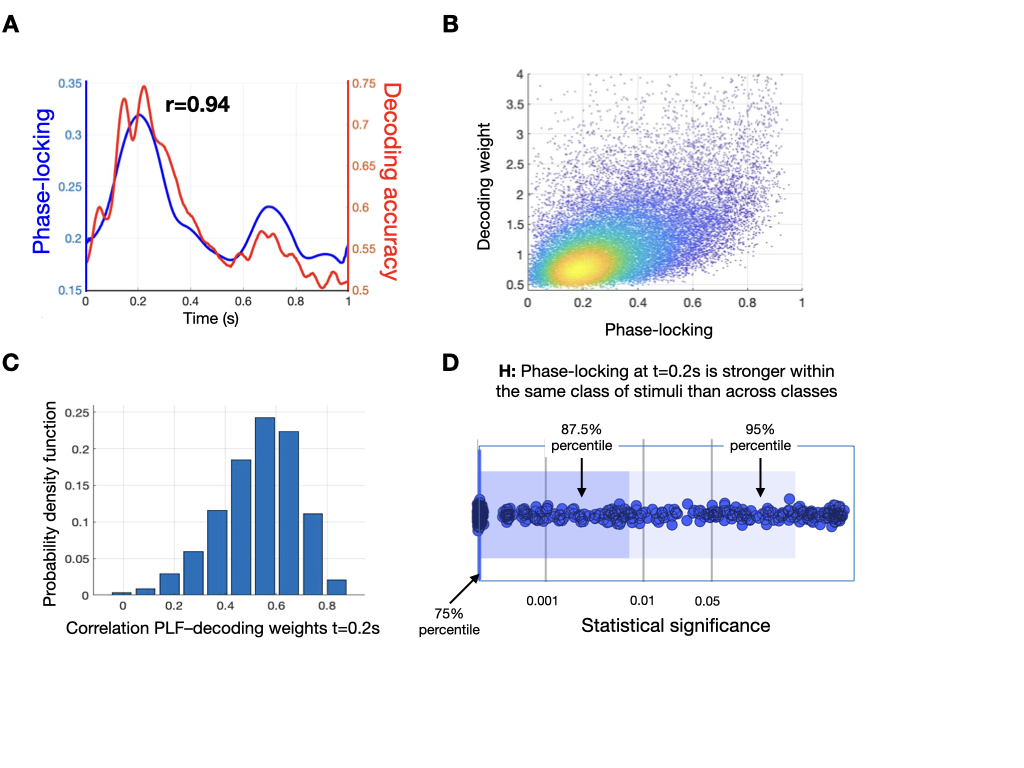
**Fig SI-5**. Oscillation-based accuracy necessitates phase-coupling across trials. **A**: The phase-locking factor (PLF; averaged across channels) and oscillation-based decoding accuracy are highly correlated across time points. **B**: Focusing on time point *t*=0.2s, scatter plots of PLF vs decoding weights for one subject. **C**: Histogram of correlations between the two measures across channels, stimuli and subjects (correlations are highly significant, p<0.001). **D**: Distribution of p-values for the hypothesis that phase-locking is stronger within trials belonging to the same stimulus than between different stimulus at *t*=0.2s, indicating that phase-resetting is highly stimulus specific; each dot represents one p-value, the vertical blue bar represent the 75% percentile (i.e. more than 75% of the p-values have the highest value of significance for the 1000 permutations we ran), and the coloured boxes represents the area containing 95% and 87.5% (lighter and darker, respectively) of the p-values.


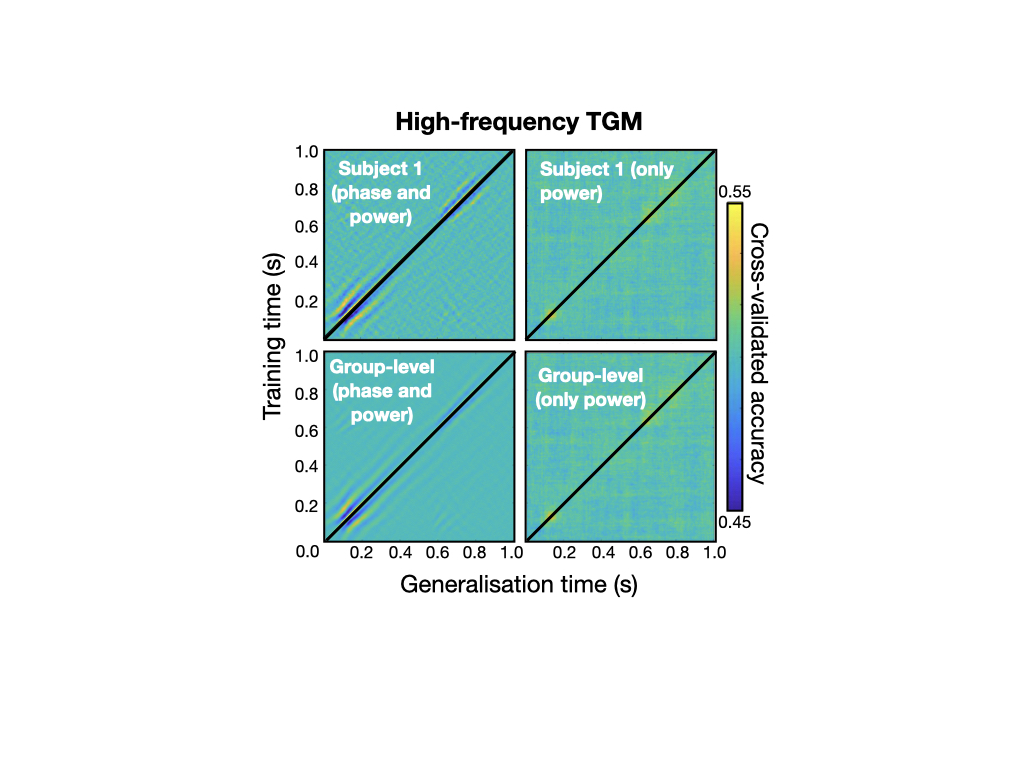


**Fig SI-6.** Temporal generalisation matrices for the remaining part of the signal, named here high-frequency (>10Hz). Results are shown for one subject (top panels) as well as at the group level (bottom panels); the left panels correspond to running decoding on the raw signal (after filtering) containing both phase and power information, while the right panels correspond to the power (i.e. without phase information).


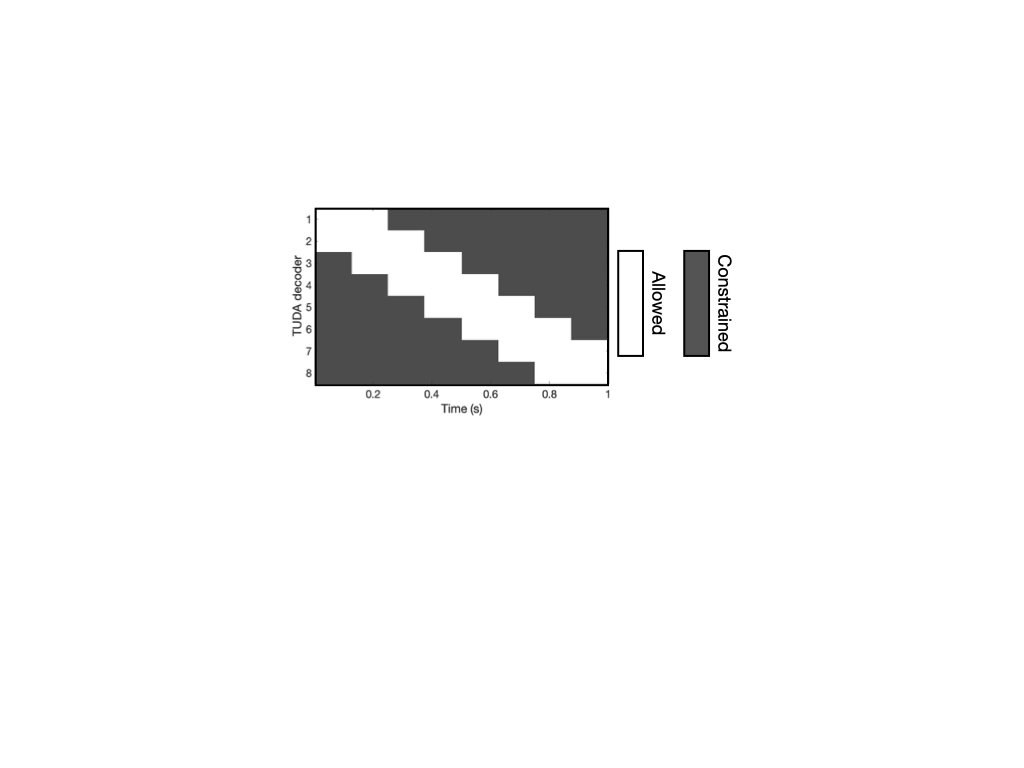


**Fig SI-7.** TUDA is imposed a sequential structure, where states are only allowed be activated at certain time points (marked in white).
